# Impact of the Gut Microbiome on the Progression of Hepatitis B Virus Related Acute-on-Chronic Liver Failure

**DOI:** 10.3389/fcimb.2021.573923

**Published:** 2021-04-06

**Authors:** Xuebing Yao, Haiping Yu, Guoyin Fan, Haihong Xiang, Lin Long, Huili Xu, Zhiguo Wu, Mingfa Chen, Wenna Xi, Zhen Gao, Cuiyun Liu, Wenlan Gong, Aoyu Yang, Ke Sun, Rongyan Yu, Junrong Liang, Baogang Xie, Shuilin Sun

**Affiliations:** ^1^ Department of Infectious Diseases, The Second Affiliated Hospital of Nanchang University, Nanchang, China; ^2^ Department of Infectious Diseases, Nanchang Centers for Disease Control and Prevention, Nanchang, China; ^3^ State Key Laboratory for Infectious Disease Prevention and Control, National Institute for Communicable Disease Control and Prevention, Chinese Center for Disease Control and Prevention, Beijing, China; ^4^ Department of Pharmaceutics, Medical College of Jiaxing University, Jiaxing, China

**Keywords:** fecal microbiome, hepatitis B virus, acute-on-chronic liver failure, biomedical indicators, metabolites

## Abstract

The relationship between the progression of hepatitis B virus-related acute-on-chronic liver failure (HBV-ACLF) and the gut microbiota is poorly understood, and an HBV-ACLF-related microbiome has yet to be identified. In this study alterations in the fecal microbiome of 91 patients with HBV-ACLF (109 stool samples), including a cohort of nine patients at different stages of HBV-ACLF, were determined by high-throughput 16S rDNA sequencing. The operational taxonomic units and Shannon indexes indicated that the diversity and abundance of the gut microbiome significantly decreased with the progression of HBV-ACLF (p <0.05). The relative abundance of the *Bacteroidetes* phylum in the microbiome was significantly reduced, whereas the abundance of potentially pathogenic bacteria, such as *Veilonella*, *Streptococcus*, *Enterococcus*, and *Klebsiella*, was highly enriched in the HBV-ACLF group compared with the healthy control group. The abundance of *Bacteroidetes* was negatively correlated with the level of serum alpha fetoprotein, and the abundance of *Veilonella* was positively correlated with serum total bilirubin (TBIL). Furthermore, the abundance of *Coprococcus* was significantly negatively correlated with the level of serum TBIL and the international normalized ratio and positively correlated with prothrombin time activity. Our findings suggest that the gut microbiota plays an important role in the development of HBV-ACLF.

## Introduction

Hepatitis B virus (HBV) infection is a major public health problem worldwide. The number of patients infected with HBV was estimated to be 93 million in China, and a small proportion of the chronic infections progress to severe hepatitis and liver failure known as HBV-associated acute-on-chronic liver failure (HBV-ACLF) (Tsai et al., 2015). However, HBV-ACLF is a devastating disease with high mortality (Wlodzimirow et al., 2012; Bajaj et al., 2014a). With the progression of liver failure, the liver tissue suffers damages from immune injury, ischemic anoxia, and endotoxemia (Zang et al., 2000; Uesugi et al., 2002; Xing et al., 2007; Chen et al., 2021). The onset and progression of HBV-ACLF have four phases (Ye and Gao, 2009): pre-rise, ascending, plateau, and recovery. In the early stage of ACLF (pre-rise phase), the body suffers from immune damage, as well as ischemia and hypoxia injury. In the middle and late stages of the ascending phase, endotoxemia causes a “second attack” to the body. In the late stages of the plateau phase and early recovery phase, the immune system is suppressed, mainly suffering from the damage from endotoxemia. Understanding the HBV-ACLF related microbiome changes may open new avenue for treatment targeted at different phases of HBV-ACLF and better management of the highly variable clinical course of HBV-ACLF (Chen EQ et al., 2015).

HBV-ACLF patients have been shown to suffer intestinal flora imbalance, with an increase in intestinal permeability and high endotoxemia (Tilg et al., 2016). The outcomes of patients with HBV-ACLF can be significantly improved if interventions are timely and appropriately provided (Ott et al., 2017). Recently, many studies have demonstrated that HBV-ACLF development is closely associated with disorders in immune function, intestinal bacterial translocation (BT), gut dysbiosis and inflammation (Verbeke et al., 2011; Wang and Li, 2015; Preveden et al., 2017; Zhang et al., 2018). A previous study showed that stimulation by translocated bacteria or bacterial products (e.g., lipopolysaccharide, lipoteichoic acid, peptidoglycans, and bacterial DNA) can activate the innate immune system and modify the adaptive immune system, resulting in inflammation, liver cell apoptosis, and progression to liver failure (Sandler et al., 2011). Early diagnosis of gut dysbiosis and correction of the intestinal flora imbalance may help prevent or delay the development of HBV-ACLF. However, the relationship of the gut microbiota with disease progression, serum biomedical indicators, and fecal metabolites remains unclear.

In this study, the HBV-ACLF related gut microbiome was investigated, and alterations in the fecal microbiome over time at different stages of the progression of HBV-ACLF were determined by high-throughput 16S rDNA sequencing. The relationships between HBV-ACLF specific microbiome and serum biomedical indicators and fecal metabolites were also analyzed.

## Materials and Methods

### Sample Collection

A total of 91 patients with HBV-ACLF in the Department of Infection Diseases of the Second Affiliated Hospital of Nanchang University were enrolled for sampling from November 2017 to April 2018. Patients with HBV-ACLF were defined in accordance with the consensus of the Asian Pacific Association for the study of the Liver (APASL) (Sarin et al., 2014; Sarin et al., 2019): with hepatitis, rapidly develops jaundice with total bilirubin (TBIL) >5 mg/dl (85 mmol/L) and coagulopathy (international normalized ratio (INR) >1.5 or prothrombin activity (PTA) <40%), and ascites or hepatic encephalopathy within 4 weeks. Severity grading and the therapeutic protocol of HBV-ACLF were performed as recommended by APASL. All individuals in the healthy control (HC) group were selected by healthy physical examination results with no history of liver and gastrointestinal diseases, no previous high blood pressure, diabetes and other systemic disease, and no history of surgery within 6 months. In addition, the HC group had no HBV infection with normal liver function and renal function. Patients with HBV-ACLF who had received antibiotic treatment 3 weeks before stool or fecal sample collection were excluded. Sampling and all subsequent steps described in the *Materials and Methods* have been conducted in accordance with the approved guidelines. Clinical information was collected in accordance with standard procedures.

All stool and blood samples were collected after the diagnosis of HBV-ACLF stages of the patients, and the corresponding clinical data were recorded to assess the grade. A cohort of nine patients was sampled continuously at each stage of HBV-ACLF after treatment (Phase 1: rising stage (ascending stage); Phase 2: plateau stage and Phase 3: recovery stage). Note that none of the patients was at pre-rise stage, as patients at this stage are usually not yet hospitalized. The stool samples of the nine cohort patients were divided into equal portions, one for fecal flora detection and the other for metabolites analysis. After collection, the fecal samples were immediately frozen and stored at −70°C before analysis. The blood samples of all patients were measured for the serum biomedical indicators: alanine transaminase (ALT); aspartate transaminase (AST); total bilirubin (TBIL); prothrombin activity (PTA); international normalized ratio (INR); WBC (white blood cells); PLT (platelet); AFP (alpha-fetoprotein) (**Table 1**).

**Table 1:** Clinical characteristics and of the subjects in this study.

Variables	Phase 1	Phase 2	Phase 3	p-Value*
Age (year)	44.9 ± 12.5	41.2 ± 13.4	48.7 ± 7.6	
Male (Number)	31 (79%)	29 (88%)	30 (83%)	
ALT (IU/l)	436.10 ± 959.54	268.67 ± 520.41	62.721 ± 61.06	0.048^*^
AST (IU/L)	375.60 ± 767.07	146.81 ± 224.43	62.78 ± 40.50	0.016^*^
TBIL (μmol/L)	280.98 ± 136.44	246.02 ± 139.36	149.15 ± 125.71	0^*^
PTA (%)	33.42 ± 7.53	52.49 ± 24.03	81.44 ± 43.79	0.043^*^
INR	1.54 ± 0.49	1.37 ± 0.41	1.27 ± 0.45	0.042^*^
WBC(×10^9^/l)	5.59 ± 2.33	6.26 ± 2.17	5.07 ± 1.85	0.075
PLT (×10^9^/l)	130.38 ± 73.02	138.79 ± 80.74	146.86 ± 94.89	0.696
Blood ammonia (μmol/l)	57 ± 32.52	65.76 ± 24.44	68.64 ± 25.44	0.496
AFP (ng/ml)	191.84 ± 273.63	223.69 ± 349.45	121.84 ± 158.10	0.454

^*^The difference is statistically significant among the groups with ANOVA.

ALT, alanine transaminase; AST, aspartate transaminase; TBIL, total bilirubin; PTA, prothrombin activity; INR, international normalized ratio; WBC, white blood cell; PLT, platelet; AFP, alpha-fetoprotein.

### DNA Extraction and Sequencing

Total genomic DNA was extracted from fecal samples using the QIAamp DNA Stool MiniKit. DNA samples were quantified using a Qubit 2.0 Fluorometer (Invitrogen, Carlsbad, CA, USA). PCR amplicons targeted with the V3 and V4 regions of 16S rRNA were generated as previously described by Kozich et al. (2013), and primer 341F/806R was used to amplify the V34 region of the 16S rRNA gene. Then, the PCR products were checked for size and specificity by agarose gel electrophoresis and purified. Finally, the amplicons were then sequenced using Illumina MiSeq in accordance with the manufacturer’s instructions (Illumina, San Diego, CA, USA) (Klindworth et al., 2013). Sequencing was performed using a 2 × 250 paired-end configuration; image analysis and base calling were conducted by MiSeq Control Software embedded in the MiSeq instrument.

### Bioinformatic Analysis

The raw reads were filtered to remove low-quality and polyclonal sequences in QIIME(Version 1.9.1) (Kuczynski et al., 2011). The filtered data were further compared with the Gold database and the chimera reads were detected using the Uchime algorithm in Usearch software(Version 8.1.1861) (Edgar, 2013). Resulting reads for each sample were clustered into operational taxonomic units (OTUs) at 97% similarity using the Uclust algorithm in QIIME (Version 1.9.1).

The representative sequence for each OTU was selected, and the taxonomic information was annotated using QIIME (Version 1.9.1) and the Greengene database (Version 13_8). The alpha diversity indices, including the observed OTUs, Chao’s richness, Simpson’s evenness, and Simpson’s reciprocal indexes, were calculated using QIIME (Version 1.9.1). A Principal Coordinate Analysis (PCoA) based on the weighted and unweighted UniFrac distances was conducted to compare all samples. Linear discriminant analysis (LDA) effect size (LEfSe) was measured using the Kruskal–Wallis rank sum test to detect features with significantly different abundances at the phylum and genus levels among the HC, Phase 1, Phase 2, and Phase 3. Spearman’s correlation coefficients between genera and clinical indices were calculated and visualized using R software (Version 3.4.1).

### ^1^H NMR Spectroscopy of Stool Specimen

Stool samples were prepared for ^1^H NMR spectroscopy as previously described (Xie et al., 2014). The metabolites were identified in the ^1^H NMR spectra by comparing their chemical shifts and coupling patterns with the corresponding values from the literature and publicly accessible databases (http://www.bmrb.wisc.edu, http://www.hmdb.ca). Total correlation spectroscopy (TOCSY), 13C-heteronuclear single-quantum correlation (13C-HSQC), statistical total correlation spectroscopy (STOCSY), and standard compounds were also utilized to identify the metabolites.

### Statistical Analysis

The Shannon and Simpson indexes were calculated with QIIME (Version 1.9.1). PCoA was performed and displayed using the ade4, cluster, fpc, and cluster Sim packages in R software (Version 3.4.1).

Data are expressed as mean ± standard deviation (SD) for continuous variables. ANOVA was performed to evaluate the difference followed by Tukey *post hoc* test. Continuous variables were compared using an independent t-test (for normally distributed ones) or nonparametric test, such as the Wilcoxon test (for those with skewed distribution) in SPSS version 11.0 for Windows (SPSS Inc., Chicago, IL, USA). Group differences in the distribution of categorical variables were analyzed using Pearson’s chi-squared test. All p values for bacterial microbiome analyses were corrected using the Benjamini–Hochberg false discovery rate (FDR) correction, and the resulting corrected values were referred to as q values. The q values lower than 0.05 were accepted as significant. Correlation between variables was tested using Spearman rank correlation and visualized by Cytoscape (Version 3.2.1).

### Data Accessibility

The raw sequence data reported in this paper have been deposited in the Genome Sequence Archive (Genomics, Proteomics & Bioinformatics 2017) in the National Genomics Data Center (Nucleic Acids Res 2021), the China National Center for Bioinformation/Beijing Institute of Genomics, Chinese Academy of Sciences, under accession number CRA003748, which is publicly accessible at https://bigd.big.ac.cn/gsa (Wang et al., 2017; C.-N. Members, and Partners, 2021).

### Ethical Approval

This study was approved by the Institutional Review Board of Second affiliated hospital of Nanchang University (KBSMC 2013-01-245-008, registered December 23, 2013). Written informed consents for all participants were obtained.

## Results

### Sample Composition and 16S Amplicon Sequencing

Among the 91 patients enrolled in our study, only nine patients had stool samples collected at all three stages of HBV-ACLF during treatment (with 27 specimens in total collected); the rest 82 patients had samples collected once per person from one of the three stages. Of the 109 stool samples, 39 were collected at the ascending stage, 34 were at the plateau stage, and 36 were at the recovery stage. Samples from 30 HC were also collected.

16S rDNA was amplified from all 139 samples and sequenced. A total of 8,055,507 reads were obtained and passed the quality filters. On average, each sample had 57,953 reads, which were assigned to 811 OTUs. Of these OTUs, 99.4% were successfully assigned at the phylum level and 55.2% at the genus level. Rarefaction curve analysis was performed to determine whether the microbiome diversity was adequately captured. For majority of the samples, the number of observed OTUs plateaued after 20,000 reads.

### Microbiota Changes in Patients With HBV-ACLF

**Figure 1:**
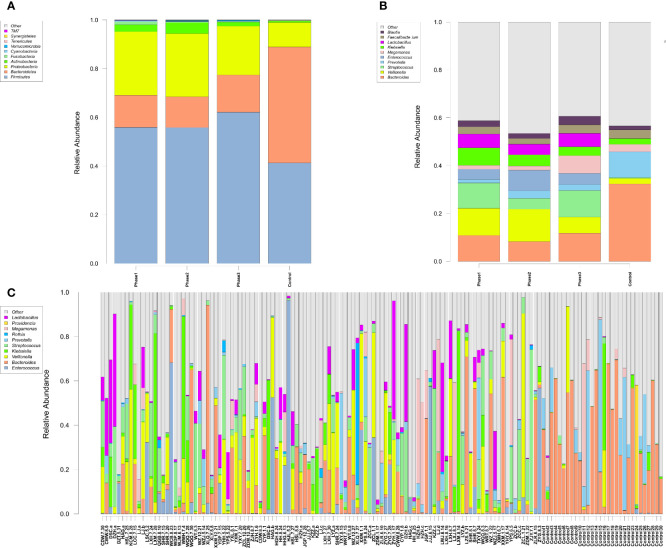
Characteristics of gut microbiota composition. **(A)** Relative abundance of microbial communities at the phylum level. Phase 1: rising stage; Phase 2: plateau stage; and Phase 3: recovery stage. **(B)** Relative abundance of microbial communities at the genus level. Phase 1: rising stage; phase 2: plateau stage; and phase 3: recovery stage **(C)** Relative abundance of all 109 samples at the top 10 genus level. (Individuals are shown along the horizontal axis, and relative taxa proportions are represented by the vertical axis. The X-axis showed the each sample, the naming principle of samples were the initials of the patients’ name added with the sampling date).

**Figure 2:**
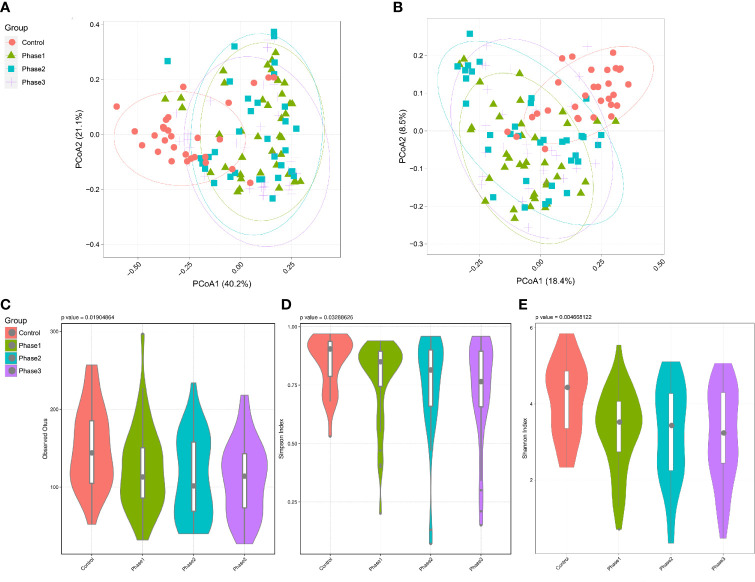
Diversity analysis of the gut microbiota of the HBV-ACLF patients. **(A)** PCoA plot based on weighted unifrac distance of the three phases of HBV-ACLF and the HC group (permutation test, p = 0.001). **(B)** PCoA plot based on unweighted unifrac distance of the three phases of HBV-ACLF and the HC group (permutation test, p = 0.001). **(C)** Operational taxonomic units (OTUs) of the three phases of HBV-ACLF and the HC group. P = 0.019 from t test. **(D)** Simpson index of the three phases of HBV-ACLF and the HC group (p = 0.033 from t test). **(E)** Shannon index indicated the community diversity of the three phases of HBV-ACLF and HC group (p = 0.0046 from t test).

**Figure 3:**
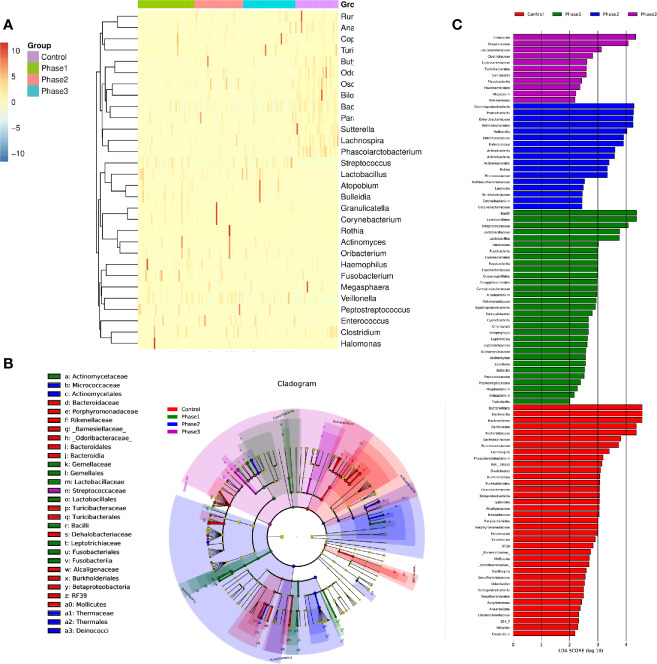
The statistically significant microbial communities associated with the HBV-ACLF. **(A)** Heatmap of 32 statistically significant genera. **(B)** Cladogram indicated the phylogenetic distribution of microbial communities associated with the three phases and the HC group; lineages with LDA values of 3.0 or higher as determined by LEfSE are shown. LEfSe (LDA Effect Size) analysis was performed to find biomarkers with significant differences (p < 0.05) between different groups. Differences are represented by the color of the most abundant class. Green sample indicates Phase 1, blue sample indicates Phase 2, purple sample indicates Phase 3, and red represents HC group. **(C)** Indicator microbial groups in the four groups with LDA scores higher than 3.0. The bar length is proportional to the LD score that indicates the significance of the genus. Differences are represented by the color of the most abundant class. Green sample indicates Phase 1, blue sample indicates Phase 2, purple sample indicates Phase 3, and red represents HC group.

**Figure 4:**
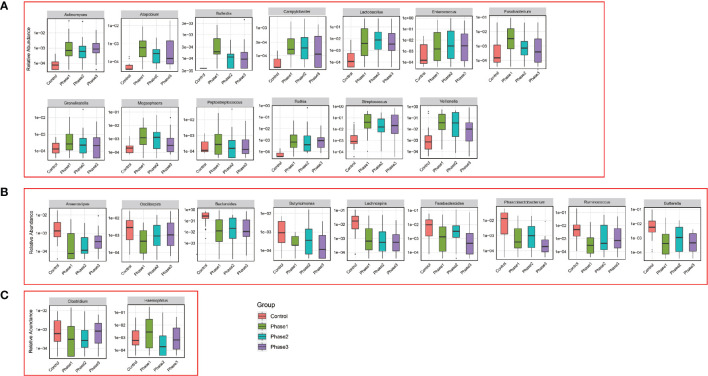
Relative abundance of genus that was statistically significant correlated with HBV-ACLF. Phase 1 (green boxplot), phase 2 (blue boxplot), phase 3 (purple boxplot), and control (red boxplot). **(A)** Relative abundance was significantly increased. **(B)** Relative abundance was significantly reduced. **(C)** Uncertain.

Among all HBV-ACLF samples, *Firmicutes*, *Bacteroidetes*, *Proteobacteria*, and *Actinobacteria* were the top four predominant phyla (**Figure 1A**). The abundance of *Firmicutes*, *Proteobacteria*, and *Actinobacteria* increased sharply, whereas that of *Bacteroidetes* decreased in the HBV-ACLF group compared with the HC group (p <0.05, Wilcoxon rank-sum test; **Figure 1A**). The intestinal flora of patients with HBV-ACLF was mainly represented by a different bacterial abundance from the HC group at the phylum level (seven phyla) and at the genus level (23 genera) (p <0.05, Wilcoxon rank-sum test; **Figures 1A, B**). The overall intestinal microbiota structure was significantly different among the three stages of HBV-ACLF. The observed operational taxonomic units (OTUs) (p = 0.019), Simpson index (p = 0.032), and Shannon index (p = 0.0047), which reflect the alpha diversity, were significantly lower in the HBV-ACLF group than in the HC group (p <0.05) (**Figures 2C, E**). The Shannon and Simpson indexes were reduced from the early phase to the recovery phase (from phases 1 to 3). At the early ascending phase (phase 1), the proportion of *Veillonella*, *Streptococcus*, *Enterococcus*, *Klebsiella*, *Lactobacillus*, and *Blautia* increased, whereas the proportion of *Bacteroidetes*, *Prevotella*, and *Megamonas* decreased significantly (p <0.05) compared with those in the HC group. In the plateau phase (phase 2), the proportion of *Veillonella* and *Enterococcus* continued to rise, and the proportion of *Bacteroidetes* decreased again. In the recovery phase (phase 3), the proportion of *Bacteroidetes* and *Megamonas* increased, and the proportion of *Enterococcus* decreased. Several genera present in the HBV-ACLF groups were absent in the HC group (e.g., *Lactobacillales* and *Streptococcus*) (p <0.05, Wilcoxon rank-sum test; **Figures 1B**, **3A**). At genus level, *Veillonella*, *Streptococcus*, *Enterococcus*, *Fusobacterium*, and *Klebsiella* were significantly more abundant in the HBV-ACLF group than in the HC group, whereas *Bacteroides*, *Ruminococcus*, *Butyricimonas*, *Lachnospira* and *Sutterella* were less abundant in the HBV-ACLF group than in the HC group (FDR-corrected p <0.05, Wilcoxon rank-sum test; (**Figures 1B**, **3A**, **4**). A significant decreasing trend was observed in the Chao1 index, Shannon’s diversity index, and OTU index between the HBV-ACLF and HC groups. PCoA was performed using UniFrac dissimilarity data. The distance between HBV-HCLF groups showed a significant separation after the pairwise comparison of weighted UniFrac distance when compared with the distances to the control group (**Figures 2A, B**) with Permutation test (p = 0.001).

### LEfSe Analysis

From the functional composition of each sample, LEfSe (LDA effect size) analysis was performed to determine the significant difference between the HBV-ACLF and HC groups (**Figures 3B, C**). Three predominant phyla (*Firmicutes*, *Proteobacteria*, and *Actinobacteria*) could be used as distinguishing biomarkers. *Firmicutes*, *Proteobacteria*, and *Actinobacteria* were significantly more abundant in the fecal microbiota of the HBV-ACLF group than in that of the HC group, whereas *Bacteroidetes* was significantly less abundant in the fecal microbiota of the HBV-ACLF group than in that of the HC group (p <0.05, **Figure 3B**).

### Correlation Analysis of Genus Abundance With Fecal Metabolites

**Figure 5:**
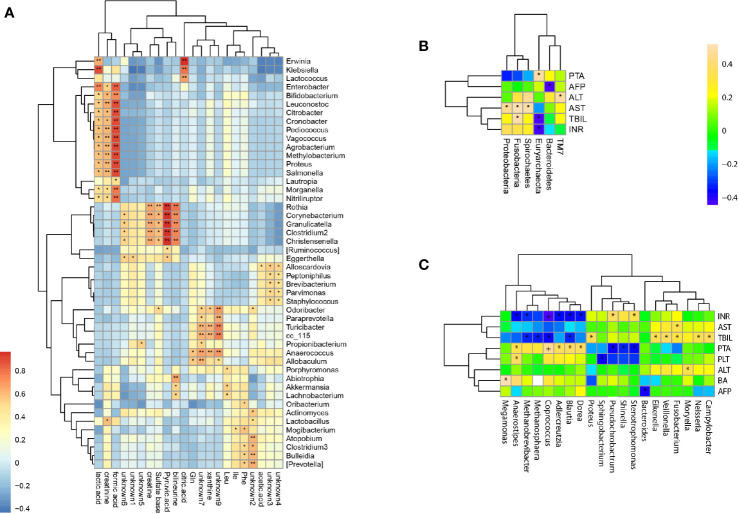
Correlations of bacterial taxon abundance with fecal metabolites and biomedical indicators. **(A)** Correlations of genus abundance with fecal metabolites (asterisk means p < 0.05). **(B)** Correlation analysis of phylum abundance with patient’s serum biomedical indicators (asterisk means p < 0.05). **(C)** Correlation analysis of genus abundance with patient’s serum biomedical indicators (asterisk means p < 0.05).

Twenty two fecal metabolites with 14 known metabolites were detected and used to determine the correlation between fecal metabolites and genus abundance. Significant correlation was found (p <0.05) and 124 genera were associated with the metabolites (**Figure 5A**). The abundance of *Christensenella*, *Clostridium*, *Corynebacterium*, *Granulicatella*, and *Rothia* were strongly positively correlated with choline, citric acid, and pyruvic acid. The abundance of *Klebsiella*, *Enterobacter*, *Bifidobacterium*, *Leuconostoc*, *Citrobacter, Cronobacter*, *Pediococcus*, *Vagococcus*, *Agrobacterium*, *Methylobacterium*, *Proteus*, *Salmonella*, *Morganella* and *Nitriliruptor*s showed a positive correlation with lactic acid, formic acid, and creatinine. The abundance of *Erwinia*, *Klebsiella* and *Lactococcus* showed a positive correlation with citric acid.

### Correlation Analysis of Genus Abundance With Serum Biomedical Indicators

The levels of serum biomedical indicators ALT, AST, TBIL, INR, and PTA showed significant differences at different stages of HBV-ACLF (p <0.05), The ALT, AST, TBIL, and INR levels showed a significant negative correlation with the increasing severities of HBV-ACLF. Only the PTA level positively correlated with the changes in the patient’s condition (**Table 1**). The serum biomedical indicators were also found to be associated with the abundance of 20 genera. The TBIL levels were significantly positively correlated with the abundance of *Veillonella*, *Fusobacterium*, *Campylobacter*, *Neisseria*, *Proteus*, and *Eikenella*, but negatively with the abundance of *Blautia*, *Coprococcus*, *Methanobrevibacter*, and *Methanosphaera*. The INR levels were positively correlated with the abundance of *Stenotrophomonas* and *Pseudochrobactrum*, but negatively with that of *Blautia*, *Dorea*, *Coprococcus*, *Anaerostipes*, *Adlercreutzia*, and *Methanobrevibacter*. The ATL and AST levels were positively correlated with the abundance of *Moryella* and *Fusobacterium* respectively. The PTA levels were positively correlated with the abundance of *Anaerostipes*, *Coprococcus*, *Adlercreutzia*, *Blautia* and *Dorea*, but negatively with that of *Shinella*, *Stenotrophomonas*, and *Pseudochrobactrum* (**Figures 5B, C**).

## Discussion

The human microbiome can influence genetic diversity, immunity, and metabolism (Zhu et al., 2010; Grice and Segre, 2012). The gut microbiome is a key determinant of intestinal inflammation and plays a significant role in host metabolic processes and host immune modulation (Hawkins et al., 2020). A strong relationship exists between the liver and the gut *via* the portal system (the venous system of the portal circulation) (Minemura and Shimizu, 2015). The gut–liver axis highlights the close anatomical and functional interaction of the gastrointestinal tract and the live (Kang and Cai, 2017). Specific microbiome patterns in stool were previously found to be present in patients with ACLF (Bajaj et al., 2014b; Zhang et al., 2019). A previous study also suggested that *Lachnospiraceae* was a biomarker of ACLF, whereas the relative abundance of *Pasteurellaceae* predicted mortality (Chen Y et al., 2015). Microbial metabolites have also been found to be associated with ACLF development (Moreau et al., 2020). In this study, diversity and abundance of the gut microbiome was found to be significantly decreased with the progression of HBV-ACLF. Bacterial taxa (phyla and genera) that were correlated positively or negatively with HBV-ACLF were identified. Further bacterial taxa that were correlated with serum biomarkers and fecal metabolites were also identified. Thus, the dysbiosis of gut microbiome detected in HBV-ACLF patients may directly contribute to the alterations of host metabolism and progression of HBV-ACLF.

As found in our study, when the balance of intestinal flora was altered, the potential pathogens of *Streptococcus*, *Veillonella*, *Klebsiella*, and *Enterococcus* were more abundant in the HBV-ACLF group than in the HC group, and the relative abundance of the beneficial bacteria *Bacteroides* was decreased. The increased abundance of pathogenic bacteria, such as *Enterococcus* and *Klebsiella*, may lead to damage the microvilli membrane of intestinal epithelial cells but also interfere with the biochemical reaction of intestinal epithelial cells. Due to dysbiosis, impairment of the intestinal micro-ecological barrier, and altered immunity status, bacterial products (such as toxins or metabolites) can reach the liver through the portal vein, where they are recognized by specific receptors, activate the immune system, and lead to a proinflammatory response (Fukui, 2015; Arab et al., 2018). *Lactobacillus* can stimulate intestinal dendritic cells to initiate immune response, reduce endotoxin, improve intestinal mucosal barrier function, and reduce hepatocyte injury. The HBV-ACLF patients enrolled in our study were all treated with *Lactobacillus* capsules to regulate the balance of intestinal microflora. Hence, the *Lactobacillus* was relatively more abundant in the HBV-ACLF group than in the HC group. Hierarchical clustering and PCoA of beta diversity discriminated the HBV-ACLF group from the HC group. The relative abundance at the phylum and genus levels was different with the development of HBV-ACLF from Phase 1 to Phase 3, with an increase in *Veillonella*, *Klebsiella* and *Enterococcus* in Phase 1 and a decrease in Phase 3; the abundance of *Bacteroides* and *Megamonas* decreased at Phase 1 and then increased in Phase 3. *Streptococcus* was abundant and enriched only in the HBV-ACLF group. Thus, the alterations of these bacterial taxa may be considered as specific biomarkers for HBV-ACLF development. A previous study showed that liver transplantation changed the gut microbiome towards increased microbial diversity, increased autochthonous bacteria (for example, *Lachnospiraceae*) and decreased potentially pathogenic bacteria (for example, *Enterobacteriaceae*) (Bajaj et al., 2017). The findings in that study were consistent with our results that the diseased liver was associated with an altered gut microbiome.

Investigation of the relationship between the gut microbiome and fecal metabolome can offer insights into the interaction of the microbiome, liver, and gut. Conversely, alteration of bacterial abundance may impact on the host and/or gut metabolism. Liver dysfunction can lead to the abnormal synthesis of important biologic signal molecules, metabolic disturbances, and alterations in serum biomedical indicators. Citric acid, pyruvic acid, lactic acid, and formic acid are the intermediate products of the tricarboxylic acid cycle, which is the common pathway of the metabolism of carbohydrates, lipids, and amino acids (Liu et al., 2017). Our results showed that the levels of citric acid, pyruvic acid, lactic acid, and formic acid in feces were strongly correlated with the abundance of 20 bacterial genera, such as *Christensenella*, *Clostridium*, *Corynebacterium*, *Granulicatella*, *RothiaKlebsiella*, *Enterobacter*, *Bifidobacterium*, *Leuconostoc*, *Citrobacter*, *Cronobacter*, *Pediococcus*, *Vagococcus*, *Agrobacterium*, *Methylobacterium*, *Proteus*, *Salmonella*, *Morganella*, *Nitriliruptor*, *Erwinia*, *Klebsiella*, and *Lactococcus.* These results suggest that HBV-ACLF could result in metabolic disorders and intestinal bacteriosis, thereby affecting physiological functions of the gust in the host.

In addition, significantly elevated levels of serum ALT, TBIL, and PTA often suggest serious liver damage and are often used to diagnose HBV-ACLF (Moreau et al., 2013; Sarin et al., 2014). HBV replication in hepatocytes could lead to a host immune reaction characterized by elevated ALT levels and may promote changes in bile acid and lipid metabolism in the liver and gut. We found that the HBV-ACLF patients’ serum biomarkers among the different stages of HBV-ACLF showed a statistical difference in the levels of ALT, AST, TBIL, INR, and PTA (p <0.05). We also found that the abundance of different genera was altered in HBV-ACLF patients and different biomarkers were correlated with the alterations of different bacterial genera. Fifteen genera were positively correlated with one of the five biomarkers (*Veillonella*, *Fusobacterium*, *Campylobacter*, *Neisseria*, *Proteus*, and *Eikenella* with TBIL; *Stenotrophomonas* and *Pseudochrobactrum* with INR; *Moryella* with ATL; and *Fusobacterium* with AST; *Anaerostipes*, *Coprococcus*, *Adlercreutzia*, *Blautia*, and *Dorea* with PTA), while 10 genera were negatively correlated with one or more of the five biomarkers (*Blautia*, *Coprococcus*, *Methanobrevibacter*, and *Methanosphaera* with TBIL; *Blautia*, *Dorea*, *Coprococcus*, *Anaerostipes*, *Adlercreutzia*, and *Methanobrevibacter* with INR; *Shinella*, *Stenotrophomonas*, and *Pseudochrobactrum* with PTA). However some genera have opposing correlation with different biomarkers, for example, *Pseudochrobactrum* was positively correlated with INR level but negatively with PTA level. Our results showed high correlations of genus abundance with serum biomarkers, such as serum ALT, AST, TBIL, and INR, in patients with HBV-ACLF. Thus, gut bacterial abnormalities may play a role in the pathogenesis and development of HBV-ACLF.

## Conclusion

High-throughput sequencing of 16S rDNA and fecal metabolite analysis were employed to investigate the impact of the gut microbiome and metabolic alterations of HBV-ACLF patients. Results showed that the diversity and abundance of the gut microbiome were perturbed in HBV-ACLF patients. Strong correlations were observed between the gut bacteria with fecal metabolites and serum biomarkers, such as serum ALT, AST, TBIL, and INR. Our findings suggest that the gut microbiota plays an important role in the pathogenesis and development of HBV-ACLF.

## Data Availability Statement

The datasets presented in this study can be found in online repositories. The names of the repository/repositories and accession number(s) can be found below: Genome Sequence Archive under accession number CRA003748.

## Ethics Statement

The studies involving human participants were reviewed and approved by the Institutional Review Board of Second affiliated hospital of Nanchang University (KBSMC 2013-01-245-008, registered December 23, 2013). The patients/participants provided their written informed consent to participate in this study.

## Author Contributions

All authors contributed to the article and approved the submitted version. XY, JL, HY, SS, and BX prepared manuscript, writing, correction, and figures design. BX, GF, HHX, LL, HLX, ZW, MC, WX, and ZG generated experimental data and wrote the manuscript. CL, WG, AY, KS, and RY conceived the work and critically reviewed the manuscript.

## Funding

This work was supported by Jiangxi Provincial key research and development project No: 20171BBG70086; and National Natural Science Foundation of China (General Project No 81860113).

## Conflict of Interest

The authors declare that the research was conducted in the absence of any commercial or financial relationships that could be construed as a potential conflict of interest.

## Supplementary Material

Supplementary Table 1 Relative abundance of all 109 samples at the top 10 genus level.
