# Supplementary material for: Impact of the Gut Microbiome on the Progression of Hepatitis B Virus Related Acute-on-Chronic Liver Failure
Source: Front Cell Infect Microbiol. 2021 Apr 6;11:573923. doi: 10.3389/fcimb.2021.573923 (PMC8056260; doi:10.3389/fcimb.2021.573923)
Supplement: Supplementary Table 1 — Relative abundance of all 109 samples at the top 10 genus level. [file Table_1.pdf]

	Enterococcus	Bacteroides	Veillonella	Klebsiella	Streptococcus	Prevotella	Rothia	Megamonas	Providencia	Lactobacillus	Other
CDW.7.30	0.002843278	0.0003412	0.20869664	0.0895443	0.207786792	0.000152	0.0029	0	3.79E-05	0.10580787	0.381871
CWW.8.9	0	0.1376905	0.00193343	0.000417	0.04094321	0.082152	0.0006	0	0	0.25843506	0.477785
DSC.a	0.031238153	0	0	0.2253393	0.060770339	0	0.0025	0	3.79E-05	0.374744105	0.30533
DZH.a	0.000113731	0.0009098	0.03070741	0	0.360489802	0.001858	0.0002	0.000113731	0	0.507619986	0.097998
DZT.5.31	0.137842141	0.0075821	0.00018955	0.0018197	0.024603837	3.79E-05	0.0048	0	0	0.000834028	0.822314
HAG.a	0	0.2248086	0.12457351	0.0159603	0.117711729	0.014141	0.0048	0	0	3.79E-05	0.497953
HSL.7.25	0	0.0132307	0.22094169	0.014368	0.248085526	0.024376	0.0005	0	0	0	0.478505
KCM.8.28	0.00098567	3.79E-05	0.55982258	0.3830465	0.012055501	0.000227	0.0028	3.79E-05	0	7.58E-05	0.040943
LCC.7.15	7.58E-05	0.2243157	0.02376981	0.3347866	0.000113731	0	0	0.092387596	0	0.000265373	0.324285
LJ.a	0	0.0003033	0.02642354	0.0170597	0.01296535	3.79E-05	0.0005	0.097240124	0	0.001440594	0.844075
LJ.b	0.001478505	0.0660778	0.34684207	0.0023504	0.051179013	0.001896	0.002	0.096633558	0	0.183903253	0.247631
LSC.5.2	0.322465691	0.0008719	0.21843961	0.0014785	0.004890439	0.000607	0.0002	3.79E-05	0	0.002312533	0.448707
LXH.7.24	0.000796118	0.1216544	0.03809993	0.0098946	0.329971946	0.000986	0.0006	3.79E-05	0	0.005572826	0.492342
LXM.7.28	0.000189552	0.0045492	0.04788081	0.7616954	0.084388506	0.010577	#####	0.005079991	0	0.001857609	0.083744
QGB.8.10	0.092501327	0.0068239	0.00132686	0.0003033	0.195465919	0.00019	0.0373	0.000682387	0	0.012851619	0.652513
SHR.7.15	0.053794829	0.0117901	0.07347032	0.0327925	0.001288953	0.000303	0	0	0	0.002805368	0.823755
SHR.7.20	0.108537418	0.0228979	0.21480021	0.013117	0.004587156	0.001099	0.0002	0.000796118	0	0.000227462	0.63371
WCB.8.23	0.682765941	0.2397832	0.00098567	0.0115248	0.005762378	7.58E-05	0.0002	3.79E-05	0	0.000379104	0.058496
WDP.8.17	0.007278793	0.0120176	0.00902267	0.003791	0.201038744	0.000531	0.0013	3.79E-05	0	0.010084161	0.754871
WJM.6.13	0.001137311	0.026234	0.00344985	0.0280537	0.02096444	0.000152	0.0002	0.036697248	0	0.000303283	0.882781
WMH.7.4	0.009401774	0.0388581	0.20210024	0.6396239	0.001895519	7.58E-05	0.004	0.074797179	0	0.000113731	0.029153
WQQ.7.28	0.000113731	0.000834	0.14155736	0.1887558	0.200470089	0.000417	0.0003	0	0	0.004056411	0.46353
WQQ.7.30	0.050989461	0.5995906	0.00610357	0.0106149	0.001326863	0.009023	#####	0	0	0.000303283	0.321973
WT.8.3	0.000151642	0.0043218	0.00447343	0.0037152	0.395443172	0	0.0007	0	0	0.055311244	0.535901
WZ.12.21	0	0.0914777	0.02589279	3.79E-05	0.289483661	0.103723	0.0016	0	0	0.131549018	0.356244
WZJ.5.14	0	0.0059519	0.03552203	3.79E-05	0.082985822	0.124308	0.0004	3.79E-05	0	0.040639927	0.710137
XLQ.7.25	0.048070362	0.8937372	0.00015164	3.79E-05	0.019561756	0	#####	0	0	0.000265373	0.038138
XLY.8.8	0.005724467	0.0857154	0.00022746	0.0280158	0.038327394	0.012473	0.0001	0.000644476	0	0.003828948	0.82493
XXM.12.21	0.000341193	0.4887785	0.03229964	0.0003033	0.00098567	0.047957	0	0	0	0.000720297	0.428615
YDP.7.15	0.1376905	0.001289	0.03552203	0.0020851	0.02991129	0	0.0008	0	0	0.149859732	0.642884
YHH.5.22	7.58E-05	0.0011752	0.33361134	0.0030328	0.378042308	0.013875	0.0516	0.003904769	0	0.000265373	0.214421
YPS.5.22	7.58E-05	0.0018955	0.00928804	0.0047388	0.020471605	0.006217	0.0019	0.0219122	0	0.211994844	0.72151
YXL.8.1	0	0.2979756	0.05591781	0.0001137	0.02498294	0.00019	0.0021	0.129312306	0	0.006748048	0.482675
YXM.7.1	0.000341193	0.0043597	0.36359845	3.79E-05	0.068541967	0.008378	0.0034	3.79E-05	0	0.065812419	0.485518
YXY.7.20	3.79E-05	0.0001896	0.24387747	3.79E-05	0.31734779	0.000341	0.0043	0	0	0	0.433884
ZCL.12.26	7.58E-05	0.3501782	0.09075745	0.0084161	0.214686481	0.007089	0.0031	0	0	0	0.325726
ZDM.12.26	0.000113731	0.1159679	0.03336113	0	0.041246493	3.79E-05	0.0002	0	0	0.005572826	0.803473

ZJH.8.7	0.001668057	0.0001137	0.34119342	0.0128895	0.023276973	0.000493	#####	0	0	0.001554326	0.618773
ZYH.1.6	0.001668057	0.1268481	0.3027902	0.0011373	0.007354614	0.065737	0.0007	0.078171203	0	0.094510577	0.321101
CDW.8.3	0	0.0011373	0.02539996	0.0197134	0.005724467	0.000114	0.0005	0.000113731	0	0.003904769	0.943438
CYP.1.1	0.000189552	0.3526803	0.03919933	0.0396543	0.020471605	0.000341	0.0002	0	0	0.05292289	0.494389
DSC.b	0.018803548	0	0.00212298	0.5827204	0.000113731	3.79E-05	0	0	0	0.002729547	0.393472
HAG.b	0.525096671	0	0.36067935	3.79E-05	0.006520585	0	0.0019	0.000113731	0	7.58E-05	0.105618
HGH.8.24	0.001440594	0.2482372	0.03654561	0.0006824	0.002539995	0.005194	0	0	0	0.006141482	0.699219
HH.8.24	0.000227462	0.0006066	0.32113883	0.0053075	0.09579953	0.002957	0.0019	0	0	0.142353476	0.429752
HHG.6.13	0.218212146	0.0001137	0.03954053	0	0.01300326	0	0.0103	0.178444158	0	0.080256274	0.460156
HJL.5.2	0.963643946	7.58E-05	0.00026537	3.79E-05	0.009629236	0	#####	0.000151642	0	0.009667147	0.016453
HSL.7.27	0.001099401	0.0171734	0.18541967	0.024528	0.079308515	0.000758	0.0013	0	0	0.151944802	0.538517
HSL.8.8	3.79E-05	0.3016908	0.0018197	7.58E-05	0.10084161	0	#####	3.79E-05	0	7.58E-05	0.595345
HZH.a	0.000568656	0.1374251	0.04992797	0.0019334	0.085753279	0.018841	#####	0.011903859	0	3.79E-05	0.693532
JGP.12.28	0.024148912	0.2428539	0.00049284	0	0.000341193	0.048184	0.003	0.000606566	0	0.000113731	0.680264
JJJ.6.7	0.000189552	0.2003564	0.00212298	0.0225946	0.008416104	0.001289	0.0004	0.000265373	0	0.000189552	0.764197
KZZ.a	3.79E-05	3.79E-05	0.01433012	0.1191902	0.119076503	0.000303	0.0003	0	0	0.007809538	0.738873
KZZ.b	0	0	0.00208507	0.2914171	0.00598984	0.000152	0	7.58E-05	0	0.003374024	0.696907
LJ.c	0.027295474	0.0212298	0.0021988	0.0001896	0.113124574	3.79E-05	0.0409	0.216695731	0	0.010387444	0.567897
LXH.7.27	0.003563576	0.236447	0.07229509	0.0966336	0.003146562	0.000152	#####	0	0	0	0.587687
LXH.7.30	0.00496626	0.1930397	0.29907499	0.132383	0.000947759	0.000986	#####	0	0	0.123587838	0.244939
LYZ.a	0	0.0017439	0.36348472	0.0035257	0.150504208	0.010463	0.0031	0.002691637	0	0.000871939	0.463644
SHR.7.24	0.042838729	0.1648343	0.29266813	0.000417	0.000265373	0.003981	#####	0.000682387	0	0.043369475	0.450868
TXY.8.15	0	0.0665706	7.58E-05	0.0185003	0.005610736	0.453977	0.0003	0.007240883	0	0.000417014	0.447342
WRT.7.15	0.002464175	0.0148609	0.00265373	0.0103874	0.022822049	0.064334	0.0024	0.002122981	0	0.027826219	0.850102
WT.8.8	0.000189552	0.3460459	0.03343696	0.0001516	0.055576617	7.58E-05	0.0001	0	0	0.007165062	0.557245
WZ.12.27	0.063727349	0.1114944	0.38327394	0	0.000909849	3.79E-05	0	0	0	0.182576389	0.25798
XLQ.7.27	0.001213132	0.026651	0.03465009	0.008871	0.059064372	0.00072	0.6392	0	0	0.023656077	0.205929
XXM.1.1	0.378952157	0.0352567	0.39915839	3.79E-05	0.012093411	0.000152	0.0024	0	0	0.024452195	0.147547
YPS.8.24	0.002464175	0.009743	0.31412541	3.79E-05	0.111570248	0.264122	0.0077	0.109560998	0	0.001213132	0.179506
ZCC.a	3.79E-05	0.0003033	0.0018197	3.79E-05	0.297255288	0.014216	0.001	0.000113731	0	0.004852529	0.68034
ZCL.1.1	0.343733414	0.0009098	0.47308363	3.79E-05	0.04094321	3.79E-05	0.0004	0.000113731	0	0.010614906	0.130108
ZDM.1.17	0	0.0106528	0.01262416	0	0.018879369	0	0	0	0	0.021874289	0.935969
ZLR.6.10	0	0.0332853	0.00151642	7.58E-05	0.013192812	0.180529	0.0001	0.054818409	0	0.000568656	0.7159
ZYG.7.27	0.074266434	0.0137615	0.45818485	0.0083782	0.001251043	0.002502	0.0001	0	0	0.037227993	0.404314
ZYG.7.28	0.222950944	0.0200925	0.02608234	0.189514	0.021684737	0.000569	0	0	0	0.006065661	0.513041
ZYH.1.17	3.79E-05	0.0038669	0.32993404	0	0.077792099	0.006558	0.0004	0.00898476	0	0.53392979	0.038517
CWW.8.28	0.000113731	0.1903859	3.79E-05	3.79E-05	0.028432785	0.118849	0.0003	3.79E-05	0	0.028319054	0.633482
CYP.1.8	0.000151642	0.0787778	0.00477671	0	0.279930245	0	0.0057	0	0	0.020850709	0.609788

DZH.b	3.79E-05	0.0081128	0.00212298	0.0001516	0.368526803	0.036849	0.0006	0.000151642	0	0.438698916	0.14478
HAG.c	0.067670028	0	0.00022746	3.79E-05	0.006785958	0	0.0009	0	0	7.58E-05	0.924255
HH.8.25	0.003449845	0.0112594	0.01091819	0.0001516	0.00894685	0.020964	0.0001	0.002350444	0	0.015505345	0.92634
HZH.b	0.000227462	0.0639548	0.00409432	3.79E-05	0.014102661	0.054667	0.0002	0.471984229	0	0.000113731	0.390666
HZH.c	0.000834028	0.0049283	0.00026537	0	0.000227462	0.035636	0	0.461520964	0	0	0.496588
JGP.1.1	0.003336113	0.4257336	0.00011373	0	0.000189552	0.001251	0.0005	0.216582	0	0	0.352339
JJJ.6.15	0.575631208	0.0001137	0.0007203	0.0026916	0.016566836	3.79E-05	0.0157	0.000492835	0	7.58E-05	0.388013
KZZ.c	7.58E-05	0.0003033	0.03343696	0.0086815	0.522708318	0.000379	0.0013	0.000151642	0	0.170520889	0.262416
LJ.d	0.058609447	0.2173023	0.00011373	0.0001516	0.00595193	3.79E-05	0.0003	0.50500417	0	0.000606566	0.211957
LWJ.6.14	3.79E-05	0.0238835	0.03442263	0.0002275	0.042611267	0.077527	0.0108	0	0	0.492304193	0.318144
LXH.8.16	0.059481386	0.0084161	0.00303283	0.0066343	0.160360907	0.000758	0.0009	0	0	0.02195011	0.738456
LXH.8.7	0.001895519	0.2574873	0.34786565	0.0031845	0.000682387	0.000531	#####	3.79E-05	0	0.260065206	0.128175
LXJ.8.3	0.003070741	0.0051179	0.00030328	0.1135037	0.000417014	0.008227	#####	0.461483054	0	0.000151642	0.40765
LXM.8.14	0.000947759	0.0093639	0.00094776	0.812192	0.003411934	0.001441	0	0.000568656	0	0.007582076	0.163545
LYZ.b	0.000113731	0.0010994	0.13124574	0.0003033	0.220221397	0.010387	0.0018	0.003601486	0	0.001402684	0.629843
LZS.5.21	0.001743877	0.7073319	0.07210554	0.014823	0.023163242	3.79E-05	0	3.79E-05	0	0.035635757	0.145121
SHR.8.1	0.02392145	0.0076958	0.23917659	7.58E-05	0.004283873	0.001706	0.0009	0	0	0.003715217	0.718515
SHR.8.7	0.121275305	0.4429828	0.02892562	0.0040185	3.79E-05	0.000341	0.0003	0	0	0.109143984	0.293009
TXY.8.24	7.58E-05	0.0022746	0.02168474	0.0416256	0.179088634	0.366821	0.0019	0.068617788	0	0.057320494	0.260596
WQQ.8.3	7.58E-05	0.0011752	0.30316931	0.1409887	0.267647282	0.001668	0.0008	0	0	0.029001441	0.255478
WRT.8.7	0.004662977	0.0053075	0.00049284	0.0779437	0.004132231	0.008985	0.0001	0.002085071	0	0.003108651	0.893169
WZ.1.22	0.001099401	0.5406399	0.06558496	0	0.126999773	7.58E-05	#####	0	0	0.001705967	0.263818
WZJ.5.17	0	0.0110698	0.01762833	0.0004928	0.03192054	0.04572	0.0035	0	0	0.262567291	0.627114
XMH.6.7	0	0.0045872	0.00090985	0.0236182	0.0189931	0.013041	0.0002	0	0	0.000151642	0.938509
XXM.1.17	0.003601486	0.6126696	0.16365911	0	0.05887482	0.000227	0.0006	0	0	0.020623247	0.139776
YHY.6.14	0.029456365	0.0266889	0.00792327	0	0.003411934	0.000303	0.0002	0	0	0.00098567	0.931041
YPS.6.5	0.045644097	0.0023125	0.00280537	3.79E-05	0.251762833	0	0.0068	0.4780878	0	0.02096444	0.191599
ZCC.b	0	0.003374	0.00041701	0.0001516	0.014785048	0.048601	0.001	0.000151642	0	0.000113731	0.931382
ZCC.c	0	0.0136856	0.04594738	0.0005307	0.320418531	0.04136	0.0016	0.000189552	0	0.003298203	0.572977
ZCL.1.21	0.074304345	7.58E-05	0.82936538	0	0.073167033	0.00019	0	0	0	3.79E-05	0.02286
ZDM.1.22	0	0.0413981	0.01853818	0	0.188869512	0	0.001	0	0	0.002464175	0.747744
ZJY.7.1	7.58E-05	0.0001896	0.00925013	0	0.731746152	0.000455	0.0187	0.000113731	0	0.002729547	0.236788
ZLR.6.15	0.024869209	0.4901054	0.01179013	0.0005687	0.013306543	0.013041	0.0009	0.001630146	0	0.001630146	0.442149
ZYG.8.3	0.567594207	0.0157328	0.01247252	0.0684282	0.001251043	0	0	7.58E-05	0	0.00098567	0.33346
Control1	0	0.3806581	0.00018955	0.0024642	0.000720297	0.137653	0	0	0	0.000151642	0.478164
Control2	0	0.0659641	7.58E-05	0.0348017	0.005079991	0.01615	0	0.132003943	0	0.008074911	0.73785
Control3	0	0.4561377	0.00284328	3.79E-05	0.00098567	0.20047	#####	3.79E-05	0	0	0.33945
Control4	0	0.6088028	0.00204716	0	0.000189552	0.000152	0	3.79E-05	0	0	0.388771

Control5	0	0.3130639	0.00394268	7.58E-05	0.036659337	0	#####	0	0	0	0.646182
Control6	0	0.2054743	0.0138752	0.0050421	0.001288953	3.79E-05	0	0	0	0.000113731	0.774168
Control7	0.000151642	0.5471984	0.3878611	0.0008719	0.000151642	0	0	7.58E-05	0	0	0.063689
Control8	0	0.601524	0.00079612	0.0007582	0.001288953	0	#####	0	0	0	0.395557
Control9	0	0.2734476	0.00026537	0.0007961	0.000341193	0.105618	0	0.007468345	0	3.79E-05	0.612025
Control10	0	0.238987	0	0.0006824	0.001402684	0.035863	#####	0	0	0	0.723027
Control11	7.58E-05	0.0178558	0.01232087	0.0004549	0.002312533	0.091326	0	0.553605277	0	0	0.322049
Control12	0	0.0388961	3.79E-05	0	0.000834028	0.606794	0	0.139889302	0	7.58E-05	0.213473
Control13	0	0.4831678	0.00113731	0	0.000417014	0	0	0.000227462	0	0.000113731	0.514937
Control14	0	0.6481538	0.00128895	0.0001137	0.002767458	3.79E-05	0	0	0	0.000568656	0.34707
Control15	0	0.1886041	0.00026537	0.0005307	0.000341193	0.687732	0	0.008871029	0	0.000682387	0.112973
Control16	0.000379104	0.0307832	0.2560088	0.4814997	0.028811889	0	#####	0	0	0.00303283	0.199409
Control17	0	0.6763212	0.00250209	0.0002275	0.000606566	0	0	0	0	3.79E-05	0.320305
Control18	0	0.4716051	0.00022746	0.0011752	0	0	0	0	0	7.58E-05	0.526916
Control19	3.79E-05	0.6947077	0.00068239	7.58E-05	0.001137311	0.001365	0	0.046781409	0	0	0.255213
Control20	0	0.2642733	3.79E-05	7.58E-05	0.000834028	0.163962	0	0	0	0	0.570817
Control21	0	0.0159224	0.00011373	3.79E-05	0.000454925	0.600121	#####	0.040488286	0	0.000568656	0.342255
Control22	0	0.2527106	0	0	3.79E-05	0.06096	0	0	0	0	0.686292
Control23	0	0.5663053	0.00360149	0.0569793	0.000530745	0.000152	#####	0	0.00030328	0	0.37209
Control24	0.022897869	0.0026537	0.00011373	0.0107286	0.007354614	0	0.0002	0	0.53707635	3.79E-05	0.418948
Control25	0.003866859	0.2691637	0.00117522	0.0090606	0.006406854	0.062325	0	0	0	3.79E-05	0.647964
Control26	3.79E-05	0.1313216	0.0003791	0.0028054	0.003715217	0.400751	0	0	0	0.000151642	0.460839
Control27	0.00394268	0.1920161	0.00034119	0.0817727	0.000644476	0.030177	#####	0	0	3.79E-05	0.69103
Control28	0	0.6008795	0.00075821	0.0016681	0.000568656	3.79E-05	0	0	0	0	0.396088
Control29	7.58E-05	0.3133672	0.00011373	0.0007582	0.004321783	0.000114	0	0	0	0	0.68125
Control30	0	0.1585033	3.79E-05	0.0089089	0.000530745	0	0	0	0	0	0.832019
